# Protocol of a randomized controlled trial of the effectiveness of physician education and activation versus two rehabilitation programs for the treatment of Whiplash-associated Disorders: The University Health Network Whiplash Intervention Trial

**DOI:** 10.1186/1745-6215-9-75

**Published:** 2008-12-24

**Authors:** Pierre Côté, J David Cassidy, Simon Carette, Eleanor Boyle, Heather M Shearer, Maja Stupar, Carlo Ammendolia, Gabrielle van der Velde, Jill A Hayden, Xiaoqing Yang, Maurits van Tulder, John W Frank

**Affiliations:** 1 Dalla Lana School of Public Health, University of Toronto, Toronto, Canada; 2 Division of Health Care & Outcomes Research, Toronto Western Research Institute, University Health Network (UHN Rehabilitation Solutions), Toronto, Canada; 3 Department of Medicine, University of Toronto, Toronto, Canada; 4 Division of Rheumatology, University Health Network, Toronto, Canada; 5 Department of Health Policy Management and Evaluation, University of Toronto, Toronto, Canada; 6 Toronto Health Economics and Technology Assessment (THETA) Collaborative, Leslie Dan Pharmacy Building, University of Toronto, Toronto, Canada; 7 Department of Community Health & Epidemiology, Faculty of Medicine, Dalhousie University, Halifax, Nova Scotia, Canada; 8 Buffalo Grove, IL, USA; 9 Department of Health Sciences, Faculty of Earth & Life Sciences, VU University, Amsterdam, Netherlands; 10 Scottish Collaboration for Public Health Research & Policy, MRC Human Genetics Unit, Western General Hospital, Edinburgh, UK; 11 Public Health Research and Policy, University of Edinburgh, Edinburgh, UK

## Abstract

**Background:**

Whiplash injuries are an important public health problem that is associated with significant disability and high health care utilization. Recent cohort studies suggest that physician care may be the most effective treatment for patients with whiplash-associated disorders. However, these findings have not been tested in a randomized controlled trial. The purpose of this study is to determine which of physician care or two rehabilitation programs of care is most effective in improving recovery of patients with recent whiplash associated disorders.

**Methods and Design:**

We designed a pragmatic randomized clinical trial. A total of 444 participants (148 in each of three arms) who reside in Southern Ontario, Canada will be recruited from a large insurer. We will include individuals who are 18 years of age or older and who are diagnosed with Grade I or II Whiplash-associated Disorders. Participants will be randomized to physician-based education and activation or one of two rehabilitation programs of care currently in use in Ontario. Our primary outcome, self-rated global recovery and all secondary outcomes (neck pain intensity, whiplash disability, health-related quality of life, depressive symptomatology and satisfaction with care) will be measured at baseline by a trial coordinator and at 6 weeks, 3, 6, 9 and 12 months follow-up by an interviewer who is blind to the participants' baseline characteristics and treatment allocation. We will also collect information on general health status, other injuries, comorbidities, expectation of recovery, work status, pain coping, legal representation, and co-interventions. The primary intention-to-treat analysis will compare time to recovery between the three interventions. This trial will have 90% power at an alpha of 0.05 to detect a 20% difference in the rate of perceived recovery at one year. Secondary analyses will compare the health outcomes, rate of recurrence and the rate of adverse events between intervention groups.

**Conclusion:**

The results of this study will provide the public, clinicians and policy makers much needed evidence on the effectiveness of common approaches used to manage whiplash-associated disorders.

**Trial registration:**

ClinicalTrials.gov identifier NCT00546806

## Background

Whiplash is the most common traffic injury, affecting 83% of people involved in motor vehicle collisions.[1] The injury leads to "Whiplash-associated Disorders" (WAD), a clinical syndrome that includes neck pain and clusters of physical and psychological symptoms.[2,3] WAD result in a significant burden of pain, disability and health care utilization.[1,2,4-7] Moreover, WAD may increase the risk of future health problems. Studies from Sweden and Saskatchewan suggest that individuals with a history of whiplash injuries may be more likely than those without a history of whiplash injuries to suffer from future episodes of neck pain, headaches, low back pain, shoulder pain, and sleep disturbances.[1,8-10]

Exorbitant health care costs, increasing disability rates and uncertainty about the most effective management of WAD have led governments, insurers and clinicians throughout the Western world to develop treatment guidelines and programs of care for the treatment of whiplash injuries.[1,2,11-15] Traditionally, these guidelines have emphasized the delivery of clinical interventions and focused less on the environment surrounding the claim process. However, it is well known that the recovery from WAD also depends on contextual factors.[1,4,16,17]

A growing body of evidence suggests that the type, intensity and timing of health care delivery are strongly and independently associated with time to recovery. Specifically, Côté et al. found that patients who made more than two visits to general practitioners, more than six visits to chiropractors, received care from general practitioner and chiropractors and those who consulted general practitioners and specialists within the first month of their injury took longer to recover than patients who visited general practitioners once or twice.[4,5] In another cohort, Cassidy et al. investigated the effectiveness of a province-wide rehabilitation program in Saskatchewan and found that patients who attended fitness training or a multidisciplinary outpatient rehabilitation program within 120 days of their injury had slower recovery than those who received usual community care.[11] Finally, a recent randomized trial compared "education and advice" by general practitioners to "education and exercises" by physiotherapists in patients with WAD lasting more than four weeks.[18] One year after the injury, patients in the general practitioner group reported levels of neck pain and headache intensity that were lower than those treated by physiotherapists.[18] Overall, this evidence suggests that the type and intensity of clinical care strongly influences the prognosis of whiplash injuries.

Despite the efforts of governments and insurers, we still do not know whether guidelines or programs of care are effective in improving health outcomes and reducing costs of patients with WAD. To date, no randomized trials have investigated the effectiveness of a coordinated and staged multidisciplinary rehabilitation program aimed at improving the health outcomes of patients with WAD. Moreover, it is not known whether rehabilitation programs are superior to physician-based education and activation in promoting better health outcomes. Therefore, there is a need for a pragmatic randomized controlled trial to investigate which program of care yields the best outcomes for patients.

Our primary objective is to determine whether education and activation by a physician is more effective than two rehabilitation programs of care ("Soft Tissue Injury Care Model" developed by AVIVA Canada and the "Pre-approved Framework Guideline for Grade I and II Whiplash Associated Disorders" developed by the Financial Services Commission of Ontario, an arm's length agency of the Ontario Ministry of Finance) in improving recovery from WAD.

Our secondary objectives will compare the effectiveness of the three interventions on reducing neck pain intensity, reducing whiplash-related disability, improving the health-related quality of life, reducing depressive symptoms, improving satisfaction with care, shorten insurance claim durations, and reducing the recurrence rate in patients with WAD.

## Methods

### Design

**Figure 1:**
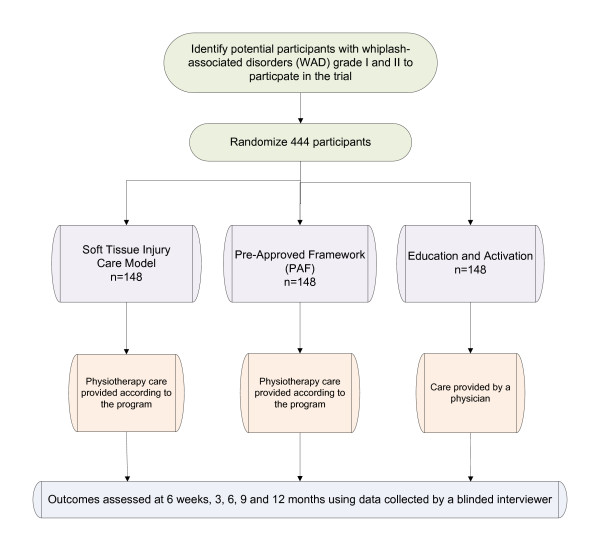
**Study Design: Process of recruitment, randomization to treatment, treatment provision, and outcomes assessment**.

We will conduct a three-arm pragmatic randomized controlled trial (Figure 1).

### Source population

Eligible for the study are individuals who: 1) are 18 years of age or older; 2) reside or work in the Greater Toronto area, Mississauga, Burlington, Cambridge or Kitchener; 3) are injured in a traffic collision; 4) make an insurance claim for physical injury to AVIVA Canada between February 2008 and March 2011.

### Recruitment

Potential participants will be identified by AVIVA claims adjusters when policy holders contact AVIVA's claim center to report an injury. The adjuster will determine whether the claimant resides or works within the catchment areas of our treatment centers and has sustained a physical injury during the motor vehicle collision. If both these criteria are met, she/he will use a standardized script to invite the claimant to learn more about the trial. Claimants who are interested will be referred immediately to the University Health Network (UHN) trial coordinator who will arrange an appointment at the nearest study clinic to assess the claimant's eligibility for the trial.

### Inclusion and exclusion criteria

All potential participants will be assessed by a trial coordinator to determine whether they meet the inclusion/exclusion criteria (Table 1). The assessment will include a history and physical examination and, if clinically indicated, diagnostic imaging.

**Table 1:** Inclusion and exclusion criteria

**Inclusion criteria**
Diagnosed with Grade I or Grade II WAD
Report an average neck pain since the accident of at least 3 on a 0–10 "Numerical Rating Scale" (NRS)
Able to give written informed consent and complete interviews in English (translators will be available to assist the participant if she/he experiences difficulty understanding specific items on the questionnaire)
Make an insurance claim for physical injury and enroll in trial within 21 days of the traffic collision

**Exclusion criteria**

Fracture/dislocation of the spine or any major bone
Head trauma associated with loss of consciousness
Past whiplash or work-related neck injury within the year prior to their current injury
Active systemic diseases (cancer, inflammatory arthritis, disorders of central nervous system)
Previous neck surgery
Received treatment from a physiotherapist or chiropractor for neck pain in the three months preceding the motor vehicle collision
Individuals who do not reside or work in the Greater Toronto, Mississauga, Burlington, Cambridge or Kitchener areas

### Randomization to treatment groups

Participants will be randomly allocated to one of the three intervention arms after they complete the consent process and answer the baseline questionnaire. If passengers from the same motor vehicle agree to participate, they will be randomized to the same treatment arm to minimize contamination and potentially minimize cross-over between treatment arms. Therefore, our unit of randomization is the motor vehicle. Central randomization was performed by the study biostatistician using statistical package, NQuery Advisor^® ^7.0.[19] To minimize the risk of unbalanced treatment assignment related to patient heterogeneity, we used block randomization which was stratified by clinical centre. The size of the blocks was randomly determined and vary between three, six, nine and 12 participants. The biostatistician provided the trial coordinators with a series of sealed opaque envelopes sequentially numbered by clinical centre. The study biostatistician is not involved in the selection of participants.

### Interventions

Participants randomly assigned to the rehabilitation programs of care will be treated at one of three University Health Network Rehabilitation Solution clinics located at the Toronto Western Hospital in Toronto, and at satellite clinics in Mississauga and Cambridge, which are areas outside of Toronto. All services will be provided by regulated health professionals including physiotherapists, kinesiologists, occupational therapists and massage therapists. Participants allocated to the physician-based "Education and Activation" arm will be referred to family physicians located at Toronto Western Hospital, the Rehabilitation Solutions clinic in Cambridge, and a private clinic in Mississauga. Health care professionals will be assigned to one treatment arm only. As recommended by the Financial Services Commission of Ontario, all participants will receive a "Getting the Facts about Whiplash" brochure. The brochure includes basic information about whiplash.[20]

### Soft Tissue Injury Care Model

The "Soft Tissue Injury Care Model" is a staged multidisciplinary rehabilitation program of care developed by AVIVA Canada. The model aims to reduce the administrative burden related to the claim process and facilitate access to timely health care. Participants randomized to the "Soft Tissue Injury Care Model" will obtain assistance from the clinic staff to complete the insurance forms necessary to initiate the claim. The program will be led by a physiotherapist and includes three integrated and sequential rehabilitation components: 1) Acute-subacute Phase; 2) Multidisciplinary Evaluation and 3) Interdisciplinary Rehabilitation.

#### Acute-subacute phase

During the first six weeks post-injury, care will be delivered by physiotherapists, and if necessary kinesiologists and massage therapists. The care consists of: 1) reassurance; 2) education; 3) home exercises, 4) physiotherapy modalities to reduce pain and 5) massage therapy. A maximum of nine treatments (including massage therapy) during the first three weeks post-collision and an additional eight sessions (including massage therapy) between the third and sixth week post-collision will be allowed. The type and frequency of treatment will be prescribed by the physiotherapist based on her/his assessment. During this phase, the physiotherapist may recommend an "in-home" or "job site" functional assessment. Finally, the physiotherapist will refer the participant to a Multidisciplinary Evaluation if she/he is not progressing as expected.

#### Multidisciplinary evaluation

Patients who require treatment beyond the acute-subacute phase will be referred for a multidisciplinary evaluation. The evaluation will be conducted by an independent team that may include a physiotherapist, physician, occupational therapist, psychologist, and kinesiologist. The purpose of the multidisciplinary evaluation is to identify barriers to recovery and recommend the appropriate treatment through interdisciplinary rehabilitation program.

#### Interdisciplinary rehabilitation

The interdisciplinary rehabilitation program has three specific goals: 1) overcome psychosocial barriers to return to function; 2) physical restoration; and 3) functional restoration. The program will include up to five weeks of daily intervention (each session can last up to 5.5 hours). The frequency, duration and type of care will be determined by the interdisciplinary team. The team may include a physician, physiotherapist, occupational therapist, and a psychologist. Moreover, the following individuals may be invited to join the team: 1) insurance claims advisor; 2) kinesiologist; 3) vocational counselor; 4) employer; 5) ergonomist; and 6) cognitive behavioral therapist. If necessary, the interdisciplinary rehabilitation program will include a jobsite assessment. The services provided during interdisciplinary rehabilitation can include: 1) education; 2) reassurance; 3) goal setting and advice on self-management; 4) psychological counseling and stress management; 5) relaxation therapy, psychotherapy and family counseling; 6) cognitive behavioral therapy; 7) instruction on pain management techniques; and 8) strength, endurance, flexibility or cardiovascular exercises.

### Pre-approved framework guideline for Grade I and II Whiplash Associated Disorders (PAF)

The PAF is a clinical management guideline that focuses on the provision of interventions to manage pain and disability through functional restoration.[20] Its use is mandated in Ontario for patients with Grade I and II WAD.[20] The treatment will be provided under the supervision of a physiotherapist and includes: 1) reassurance; 2) education; 3) home exercises, and 4) encouragement to resume normal activities of daily living. Additionally, the physiotherapist may prescribe the following interventions: 1) exercise and functional activities; 2) mobilization and manipulation; 3) pain management modalities (including massage therapy); and 4) coping skills education. The frequency and number of visits is based on the clinical judgment of the physiotherapist but cannot exceed 10 visits within the first three weeks and nine visits three to six weeks post-collision.

Participants with significant functional limitations will be eligible for a functional assessment of their home, work or school environment. Based on the assessment, occupational therapists will develop an intervention that may include: 1) recommendation to use aids or devices; 2) minor modifications at the work, home, or school environment; 3) instructions on adaptive strategies or alternate approaches to fulfill functional tasks; and 4) specific functional activities to increase tolerance.

Participants who report significant improvement in the first six weeks of care, but who have not recovered, can receive up to four additional treatments over a two-week period. Participants who have not recovered will be re-evaluated and a new plan of management will be developed by the physiotherapist in charge of the care.

### Physician-based education and activation

The education and activation intervention program is designed to promote self-care and early return to normal activities of daily living. Participants randomized to education and activation will be under the care of a family physician.

#### Initial visit

The intervention includes reassurance, education about the favorable prognosis of WAD, encouragement to resume activities of daily living, recommendation to do home exercises (stretching) and, if indicated, prescribe pain relief modalities (heat/ice, acetaminophen or a Cox-2 selective non-steroidal anti-inflammatory drug (NSAID) or non-selective NSAID with or without gastroprotection). At the end of the initial visit, the physician will determine whether a follow-up appointment is necessary or if the participant should be discharged. The participant will be informed to contact the physician should their complaint persist or worsen.

#### Follow-up visits

If follow-up visits are necessary, the participant will be re-assessed and the intervention described above will be repeated and adapted to the participant's status. Participants who have not recovered six weeks after the injury or have improved but require more care will be referred for a multidisciplinary evaluation and enter the interdisciplinary rehabilitation (described above under Soft Tissue Injury Care Model).

### Data collection and follow-up

Table 2 summarizes the data to be collected during the trial. We will follow-up participants at six weeks, and three, six, nine and 12 months post-injury. All follow-up interviews will be conducted by a research assistant either in person at the study clinic or by telephone. The study research assistant will be blind to the treatment allocation and to the baseline characteristics of participants. However, because most outcomes are self-reported by the participants, the research assistant will not be blind to their outcomes status.

**Table 2:** Measures used at baseline and follow-up interviews

**Measures**	**Baseline**	**6 Weeks**	**3 Months**	**6 Months**	**9 Months**	**12 Months**
Accident information	x					

Past history of neck pain and whiplash	x					

Numerical rating scale	x	x	x	x	x	x

Health care after accident	x					

Comorbidity questionnaire	x					

Acute 36-item short-form health survey (V2)	x	x	x	x	x	x

Work status	x	x	x	x	x	x

Expectation of recovery	x	x	x	x	x	x

Whiplash disability questionnaire	x	x	x	x	x	x

Center for epidemiological studies-depression scale (CES-D)	x	x	x	x	x	x

Vanderbilt pain management inventory	x	x		x		

Health state question	x	x	x	x	x	x

Socio-demographic characteristics	x					

Whether a lawyer or paralegal is involved in the claim	x	x	x	x	x	x

Global perceived recovery question	x	x	x	x	x	x

Satisfaction with care and treatment outcome		x	x	x	x	x

Co-interventions		x	x	x	x	x

### Outcomes

#### Primary outcomes

##### Time to recovery

Our primary outcome is time to recovery measured by the global self-perceived recovery question. The global perceived recovery question is a reliable, valid and responsive measure of health status in patients with musculoskeletal disorders.[21] This ordinal transition scale has been used in previous randomized trials and cohort studies of neck pain.[11,22,23] In patients with WAD, we have previously reported that perceived recovery is consistently associated with less neck pain, better physical functioning and fewer depressive symptoms.[11]

At each follow-up interview, participants will be asked: "How well do you feel you are recovering from your injuries?" Participants will select between one of the seven following choices: 1) completely better; 2) much improved; 3) slightly improved; 4) no change; 5) slightly worse; 6) much worse and 7) worse than ever. Participants who respond to be "completely recovered" or "much improved" will be considered recovered. Time to recovery will be measured as the number of days between the date of injury and the first follow-up date at which a participant reports to be "completely recovered" or "much improved".

#### Secondary outcomes

##### Neck pain intensity

Neck pain intensity will be measured at baseline and at each follow-up with the 11-point numerical rating scale (NRS). The NRS is a global measure of pain intensity anchored by two extremes of pain intensity ranging from 0 (referring to "No pain") to 10 (referring to "Pain as bad as it could be"). The NRS has good short-term test-retest reliability with correlation coefficient ranging from 0.95–0.99 when re-administered within 24 hours.[24] The NRS has good construct validity and can distinguish between various levels of pain in subjects with chronic post-operative pain.[24,25] It correlates well with other instruments used to measure pain.

##### Whiplash disability

Disability will be measured with the Whiplash Disability Questionnaire (WDQ).[26] The WDQ is a 13-item modified version of the Neck Disability Index. It includes 13 Likert scales scored from 0 to 10 with higher global scores indicating more disability. The instrument has adequate face validity, discriminate validity and good internal consistency (Cronbach's alpha = 0.96). [26,27] Pinfold et al. (2006)[26] reported that the WDQ has no substantial ceiling or floor effect. In patients with chronic stable symptoms, the WDQ has excellent short-term (24 hours) and medium-term (one month) reproducibility (Intra-correlation coefficient = 0.90, and 0.86, respectively).[28] The WDQ is a responsive scale with a minimal detectable change of 15 points.[28]

##### Health-related quality of life (H-RQoL)

We will use the Medical Outcomes Study Short-Form Health Survey version two (SF-36) to measure health-related quality of life. The SF-36 has 36 items which measure the H-RQoL of a subject. Two summary scores can be computed: the physical component score and the mental component score. There are eight individual scales: physical functioning, role physical, bodily pain, general health, vitality, social functioning, role emotional and mental health. The questionnaire has been shown to have excellent reliability demonstrated with internal consistency and test-retest methods. The SF-36 is a valid and reliable measure for clinical and general populations with a reported intra-class correlation coefficient of 0.85. [29-31] Further, in a study of injured workers with musculoskeletal conditions it was shown to be the instrument that is the most responsive to change.[32]

##### Depressive symptomatology

Depressive symptomatology in the previous week will be measured with the Center for Epidemiological Studies-Depression Scale (CES-D). The CES-D is a widely used 20-item self-report scale designed to measure current level of depressive symptomatology in population-based epidemiologic research.[3,33,34] It has good test-retest reliability and internal consistency and possesses good factorial and discriminant validity.[33,35-42] The CES-D is scored from 0 to 60 with higher scores indicating greater depressive symptomatology.[33,36]

##### Satisfaction with care and satisfaction with treatment

Two global questions will be used to quantify satisfaction with care and satisfaction with treatment outcome. Satisfaction with care will be measured by asking participants the following question: "All things considered, how satisfied are you with the care you received?" Similarly, satisfaction with treatment outcome will be measured by asking "All things considered, how satisfied are you with the results of your treatment?".[43,44] Participants will be asked to rate their satisfaction on a Likert scale ranging from 1 = extremely satisfied to 7 = extremely dissatisfied.

##### Time on insurance benefit

We will measure the number of days between the date of injury and the date corresponding to the closure of the insurance claim. Claim closure corresponds to the end of treatment, the attainment of maximal medical improvement, the termination of income replacement benefits or the date of payment of the last outstanding bill incurred by the claimant (e.g., eyewear). Claim closure dates will be provided by AVIVA Canada. We have previously validated time on benefit as a marker of time to health recovery in a population of whiplash patients from Saskatchewan and found that claimants who close their claims have significantly lower levels of neck pain, better physical functioning and no depression compared to claimants who have not closed their claim.[16]

##### Recurrence

A recurrence will occur when a participant reports to have recovered, but reports at any subsequent follow-up on the self-perceived recovery question to be: 1) slightly improved; 2) unchanged; 3) slightly worse; 4) much worse, or 5) worse than ever.

##### Co-interventions

We will measure co-interventions by asking participants to self-report the type health care provider consulted beyond those involved in delivering the trial interventions. Finally, we will ask participants if they have used medications for their whiplash injuries.

### Statistical Issues

#### Sample size

Our sample size was selected to detect a difference of 20% in the rate of recovery at one year using a log rank test, a power of 90% and a two-tail significance level of 0.05.[19] Based on these parameters, we need 114 participants per group. Assuming a 30% loss-to follow-up per arm we aim to enroll a total of 444 participants (148 per intervention arm).

It is common for vehicles involved in traffic collisions to have multiple passengers. Early in our trial, we observed that some participants enrolled in the study had a spouse or relative with them in the car at the time of the collision that was also eligible for the trial. To prevent contamination bias and promote treatment compliance, we decided to change the randomization unit from the individual to the motor vehicle. Consequently, all passengers from the same vehicle are randomized to the same study arm. We have observed that less than 5% of vehicles have multiple passengers. Therefore, our original sample size of 444 participants remains valid.

### Statistical analysis

Our primary analysis focuses on studying treatment policy and therefore will be conducted according to the intention-to-treat principle, that is, participants will be analyzed according to their randomized intervention group whether or not they received the intervention. However, we are also interested in understanding the impact of treatment self-selection and protocol deviations on the primary and secondary outcomes. To achieve this goal, we will also analyze the trial using the "as treated" (participants will be analyzed according to the treatment they received) and "per-protocol" (the analysis will be restricted to participants who were compliant with the treatment protocol) approaches.

### Primary outcomes: self-perceived recovery

Our primary analysis will compare the time to recovery across intervention arms using the Kaplan-Meier method. We will report the median time to recovery and 95% confidence interval (CI).[45] We will use mixed-effect Cox proportional hazards model to measure the effectiveness of the physician-based education and activation intervention and of the Soft Tissue Injury Care Model relative to the PAF. [45-49] Effectiveness will be reported as the Hazard Rate Ratio (HRR) and 95% confidence interval (CI). Participant characteristics that vary between the three interventions at baseline will be included in a multivariable Cox proportional hazards model to control for their confounding effect. Covariates added to the crude Cox Model that change any of intervention regression coefficients by 10% percent or more will be retained as confounders in the adjusted model.[50] The observations of participants who drop out of the study before they report to have recovered will be censored on the date of their last completed follow-up interview. No imputation methods will be used to deal with missing outcomes.

### Secondary outcomes

To analyze the continuous secondary outcomes (neck pain intensity, whiplash disability, health-related quality of life, depressive symptomatology, satisfaction with care), we will first compute the intervention-specific mean, standard deviation and median at each follow-up interval. Second, we will build ordinary least-square (OLS) models using generalized estimating equation (GEE) to account for the autocorrelation present in the outcomes.[51] Third, we will test whether the intervention effects are constant throughout the follow-up periods.[51] Fourth, we will test whether imbalances in the distribution of the baseline covariates confound the intervention effects. Covariates added to the crude linear model that change any of intervention regression coefficients by 10% percent or more will be retained as confounders in the adjusted models.[50] The intervention effects will be reported as the mean differences and 95% for each follow-up interval.

We will analyze time to claim closure using the Kaplan-Meier method and the Cox proportional hazards model as described above. Effectiveness will be reported as the HRR and 95% CI.

To analyze the recurrence rate, we will first compute the intervention-specific incidence (95% CI) of recurrence. Second, we will use Poisson regression and GEE to account for the autocorrelation present in the report of self-perceived recovery.[51] In the Poisson model, the number of recurrences will be used as the outcome variable. Third, we will test whether imbalances in the distribution of the baseline covariates confound the intervention effects. Covariates added to the crude Poisson model that change any of intervention regression coefficients by 10% percent or more will be retained as confounders in the models.[50] We will present the intervention effects as relative risks (RR) and 95% CI.

### Pilot study

We conducted a pilot study from February 1 – 25, 2008. The purpose of the pilot study was to test the feasibility and implementation of the trial. Specifically, we aimed to: 1) determine the referral rate from AVIVA Canada; 2) determine the participation rate; 3) test the administration of the baseline questionnaire; 4) ensure that the interventions can be delivered in a timely fashion and according to the protocol and 6) determine the 6- week follow-up rate.

The trial coordinators received 41 referrals during the pilot study for a referral rate of 1.6 calls per day. Of those, 36 were eligible and 12 consented to participate (participation rate = 33.3%). Our baseline and follow-up questionnaires were well received by participants. The six week follow-up rate was 58.3% (7/12). One participant dropped out of the study to receive care at another clinic and four participants could not be reached to complete the follow-up interview. The interventions were delivered without any difficulties.

### Protection of human subjects and assessment of safety

#### Protection of human subjects

The study protocol was approved by the University Health Network Research Ethics Board (07-0623-A).

#### Adverse events

We will measure the presence of adverse events that may be associated with the treatments at each follow-up interview. We defined adverse events as an unintended sign or symptom of the intervention. These include: increase in neck pain, stiffness, radiating pain/discomfort in arms, arm weakness, leg weakness, feeling tired, headache, dizziness, depression, anxiety or other physical discomfort. We will compute the intervention-specific incidence (95% CI) of each adverse event listed above. The intervention-specific cumulative number of visits will be used as the denominator. Any adverse event that is life-threatening or associated with significant disability will be reported to the University Health Network Research Ethics Board.

## Competing interests

The authors declare that they have no competing interests.

## Authors' contributions

PC, JDC, EB, SC, CA, MV, JAH, JWF, GV, XY participated in the conception and design of the trial. The analysis was designed by PC, JDC, EB and XY. PC, EB, HMS and MS participated in the implementation of the pilot study. All authors participated in drafting the manuscript and approved its final version.
